# A Transformer-Based Detection Network for Precision Cistanche Pest and Disease Management in Smart Agriculture

**DOI:** 10.3390/plants14040499

**Published:** 2025-02-07

**Authors:** Hang Zhang, Zimo Gong, Chen Hu, Canyang Chen, Zihang Wang, Boda Yu, Jingchao Suo, Chenlu Jiang, Chunli Lv

**Affiliations:** China Agricultural University, Beijing 100083, China

**Keywords:** cistanche pest and disease detection, Transformer-based detection network, dynamic loss balancing, smart agriculture, disease management

## Abstract

This study focuses on pest and disease detection in cistanche, proposing a Transformer-based object detection network enhanced by a bridging attention mechanism and bridging loss function, demonstrating outstanding performance in complex agricultural scenarios. The bridging attention mechanism dynamically fuses low-level details and high-level semantics, significantly improving detection capabilities for small targets and complex backgrounds. Experimental results show that the method achieves an average accuracy of 0.93, a precision of 0.95, a recall of 0.92, and mAP@50 and mAP@75 scores of 0.92 and 0.90, outperforming traditional self-attention mechanisms and CBAM modules. These results confirm the method’s ability to overcome challenges such as unclear disease features and small target sizes, providing robust support for precision pest detection. The research contributes to smart agricultural disease management and the sustainable development of cistanche cultivation while laying a solid foundation for future agricultural intelligence applications.

## 1. Introduction

Cistanche is a medicinal plant widely cultivated in the northwestern regions of China, particularly in the Bayannaoer area of Inner Mongolia. Its roots, rich in alkaloids and various amino acids, have been extensively used in traditional Chinese medicine [1,2,3]. In modern industrial applications, cistanche extracts are also important ingredients in health products, and the market demand has been increasing year by year [4,5,6]. However, the efficient cultivation and production of cistanche face serious challenges from diseases, which not only affect plant growth and development but also severely reduce medicinal value and yield, causing significant economic losses to the economic forestry industry in Bayannaoer.

In traditional cistanche disease management practices, farmers typically rely on experience for disease identification and treatment [7,8]. This method, based on personal experience and intuitive judgment, is inefficient and prone to diagnostic errors, which can lead to improper disease control or excessive pesticide use, ultimately affecting plant growth and soil health [9]. Moreover, the wide cultivation areas and complex geographical environments of cistanche make traditional disease management methods even more challenging, making it difficult to achieve precise and efficient disease control.

With the development of information technology, especially the successful application of deep learning in image processing and pattern recognition, new solutions for agricultural disease detection have emerged [10,11,12]. Deep learning models can automatically learn disease features from large amounts of data, enabling an efficient and accurate identification of cistanche diseases. This technology is not limited by personal experience or subjective judgment, significantly improving the accuracy and responsiveness of disease detection and offering possibilities for precision agriculture and intelligent disease management [13]. For instance, Chen et al. [14] proposed a Transformer-based model for generating diseased tomato leaf images for data augmentation using a cyclic consistency generative adversarial network. Kumar et al. [15] proposed an accurate method for detecting rice leaf diseases and evaluated it. They used the DenseNet-Bi-FAPN with YOLOv5 model, integrating the YOLOv5 network with DAIS segmentation and Bi-FAPN networks. Their experimental results showed an accuracy of 94.87%, and the proposed method helped farmers detect rice leaf diseases at an early stage.

To detect diseases efficiently, Chitraningrum et al. [16] used convolutional neural network algorithms, namely YOLOv5 and YOLOv8, to detect maize leaf diseases. The experimental results showed that YOLOv8’s mAP50 reached 0.965, indicating a higher detection rate. Sethy et al. [17] proposed a new method for detecting rice false smut disease based on Faster R-CNN. The Faster R-CNN process includes region proposal generation and object detection, both of which are performed in the same convolutional network. Due to this design, the object detection speed is faster, but in some complex cases, it may lead to detection failure. In response, Yu [18] described a composite method that integrates deformable convolution and Faster R-CNN networks. The deformable convolution enhances the model’s spatial sampling ability, enabling it to capture deformed information in the image, while Faster R-CNN provides a robust framework for object detection. Zhao et al. [19] proposed a new Faster R-CNN architecture to address the issue of complex backgrounds and small disease spots in strawberry disease images in natural environments. Their multi-scale feature fusion network, consisting of ResNet, FPN, and CBAM (Convolutional Block Attention Module) blocks, effectively extracts rich strawberry disease features. They constructed a dataset of strawberry leaves, flowers, and fruits, and the experimental results showed that the model could effectively detect healthy strawberries and seven types of strawberry diseases under natural conditions, with an mAP of 92.18% and an average detection time of only 229 ms, improving the management of strawberry growth for farmers. However, the dataset used for detection was small, and the model’s applicability could not be guaranteed.

This study focuses on the cistanche planting area in Bayannaoer as the research subject, developing a deep learning-based cistanche disease detection model. The model employs the latest object detection techniques, combined with region-specific disease features, and trained on a large amount of real-world image data, enabling the rapid and accurate detection of cistanche diseases. Additionally, the model introduces a bridging attention mechanism to enhance its learning ability of key disease features, further improving detection accuracy and robustness. Furthermore, a specialized bridging loss function is designed to optimize the model’s performance in complex backgrounds, ensuring its efficiency and reliability in practical deployment.

## 2. Related Work

### 2.1. Single-Stage Object Detection Networks

Single-stage object detection networks have been widely used in various real-time application scenarios due to their advantages in detection speed and simplified training process [20,21]. Unlike traditional two-stage object detection networks, single-stage networks do not require generating candidate regions. Instead, they directly predict the object classes and locations at the output layer of the network, significantly improving processing speed [22,23,24]. The basic principle of single-stage object detection networks involves dividing an image into multiple grids with each grid responsible for detecting objects that fall within it. For example, in the YOLO network, the input image is divided into an S×S grid, where each grid predicts *B* bounding boxes and the corresponding class probabilities for those boxes [25,26]. Each bounding box consists of five predicted values: the *x* and *y* coordinates of the box’s center, the width *w* and height *h* of the box, and the confidence score *C* indicating the presence of an object within the box. The confidence score is defined as $P_{\mathrm{obj}}\cdot {\mathrm{IoU}}_{\mathrm{pred}}^{\mathrm{truth}}$, where $P_{\mathrm{obj}}$ represents the probability of the object being present in the grid, and ${\mathrm{IoU}}_{\mathrm{pred}}^{\mathrm{truth}}$ represents the Intersection over Union (IoU) between the predicted box and the ground truth box. The objective function of the YOLO network consists of three components: location error, confidence error, and classification error. The location and confidence errors are computed only when the grid contains an object, while the classification error is computed across all grids. The specific objective function can be represented as

(1) $$\begin{array}{cc} \; & \lambda_{\mathrm{coord}}\sum_{i=0}^{S^{2}}\sum_{j=0}^{B}1_{ij}^{\mathrm{obj}}\left[{\left(x_{i}-{\hat{x}}_{i}\right)}^{2}+{\left(y_{i}-{\hat{y}}_{i}\right)}^{2}\right]\\ & +\lambda_{\mathrm{coord}}\sum_{i=0}^{S^{2}}\sum_{j=0}^{B}1_{ij}^{\mathrm{obj}}\left[{\left(\sqrt{w_{i}}-\sqrt{{\hat{w}}_{i}}\right)}^{2}+{\left(\sqrt{h_{i}}-\sqrt{{\hat{h}}_{i}}\right)}^{2}\right] \end{array}$$

(2) $$\sum_{i=0}^{S^{2}}\sum_{j=0}^{B}1_{ij}^{\mathrm{obj}}{\left(C_{i}-{\hat{C}}_{i}\right)}^{2}+\lambda_{\mathrm{noobj}}\sum_{i=0}^{S^{2}}\sum_{j=0}^{B}1_{ij}^{\mathrm{noobj}}{\left(C_{i}-{\hat{C}}_{i}\right)}^{2}$$

(3) $$\sum_{i=0}^{S^{2}}1_{i}^{\mathrm{obj}}\sum_{c\in \mathrm{classes}}{\left(p_{i}\left(c\right)-{\hat{p}}_{i}\left(c\right)\right)}^{2}$$

Here, $1_{ij}^{\mathrm{obj}}$ is an indicator function, which equals 1 if the *j*-th bounding box in grid *i* predicts an object and 0 otherwise; $1_{ij}^{\mathrm{noobj}}$ indicates that the *j*-th bounding box in grid *i* does not contain an object. The parameters $\lambda_{\mathrm{coord}}$ and $\lambda_{\mathrm{noobj}}$ are the weights that regulate the coordinate prediction and the confidence prediction for regions without an object, respectively. In the application of cotton aphid disease detection, single-stage networks can quickly assess the disease condition across large agricultural fields. Especially when using drones for large-scale monitoring, networks like YOLO can achieve real-time disease detection and localization [27]. However, since the features of cotton aphid disease are often small and the background in natural environments is complex, this increases the challenge for single-stage networks in detecting small objects [28].

### 2.2. Two-Stage Object Detection Networks

Two-stage object detection networks are renowned for their high accuracy in object detection, especially for applications that demand extremely high precision. Faster R-CNN, one of the representative networks of this category, greatly improves detection efficiency and accuracy by introducing a Region Proposal Network (RPN) that automatically extracts high-quality candidate regions [29,30,31]. The core of Faster R-CNN lies in its two-stage processing flow. In the first stage, the network automatically generates region proposals that may contain objects in the image using RPN. RPN is a convolutional network that scans each position of the input image, using a sliding window approach to extract features, and predicts multiple candidate regions, called anchors, at each window position. Each anchor corresponds to several preset bounding boxes, known as anchor boxes, and the network needs to predict which of these anchor boxes contain objects [32,33,34]. In the second stage, for each candidate region proposed by RPN, Faster R-CNN uses the RoI (Region of Interest) pooling layer to extract fixed-size region features from the feature map, which are then passed through subsequent fully connected layers for more detailed classification and bounding box regression. This stage not only determines the object category within the candidate region but also fine-tunes the position of the bounding box [35]. The relevant computation can be expressed as

(4) $$\mathrm{RoI}\left(R\right)={c_{j},\;b_{j}}_{j=1}^{M}$$

where *R* represents the candidate regions passed from RPN, $c_{j}$ is the object category in the candidate region, and $b_{j}$ is the corresponding bounding box coordinates. A potential advantage of Faster R-CNN in the application of cotton aphid disease detection is its excellent region proposal capability and precise bounding box regression, which can effectively handle the complex variations of disease features in natural environments [36,37]. However, Faster R-CNN has a high computational complexity, especially in scenarios that require real-time processing, where its speed becomes a limiting factor [38,39].

### 2.3. DETR: Transformer-Based Object Detection Network

DETR (Detection Transformer) represents a significant innovation in the field of object detection by applying the Transformer architecture to detection tasks, which significantly alters the traditional methodologies of object detection [40]. Unlike traditional region proposal-based object detection methods, DETR eliminates the need for complex region proposal networks and non-maximum suppression (NMS), offering a simpler and more direct solution [41,42]. The core of DETR lies in using the Transformer’s encoder–decoder structure to process image data. In this architecture, the encoder first converts the input image into a series of feature representations that capture the global information of the image. Then, the decoder gradually generates predictions using self-attention mechanisms, where each result corresponds to an object in the image. The encoder receives feature maps from the convolutional neural network as input, projects them into a higher-dimensional space, and processes these features through a series of self-attention layers and feedforward networks. This process can be expressed by the following formula:

(5) Encoder(z)=LayerNorm(z+MultiHeadAttention(z, z, z))

(6) Updatedz=LayerNorm(z+FeedForward(z))

where z is the representation of the input feature map, and MultiHeadAttention and FeedForward represent the multi-head attention mechanism and the feedforward network, respectively. These layers are repeated multiple times in the Transformer to enhance the model’s expressive ability. In the decoder part, DETR uses a set of learned object queries, which interact with each self-attention layer in the decoder to progressively refine the information about each predicted object. The key here is that each query independently predicts the category and bounding box of an object. The output of the decoder is passed through a linear layer to generate the final class probabilities and bounding box coordinates. This process can be expressed as

(7) Decoder(q, z)=LayerNorm(q+MultiHeadAttention(q, z, z))

(8) Updatedq=LayerNorm(q+FeedForward(q))

where q represents the object queries, and z is the feature map representation passed from the encoder. In the application of cotton aphid disease detection, this architecture allows DETR to precisely distinguish disease areas directly from complex backgrounds, making it especially suitable for multi-object and complex background disease detection tasks [43,44]. However, DETR, through its global attention mechanism, better understands the relationship between each element in the image and the overall context, thus significantly improving the accuracy and efficiency of disease detection [45,46].

## 3. Materials and Method

### 3.1. Dataset Collection

In the study of cistanche disease detection models, dataset collection is a critical step. The dataset used in this research primarily comes from two sources: field collection and online sources, ensuring diversity and broad representation in the data. Field-collected images were sourced from Dengkou County in Bayannur City, Inner Mongolia Autonomous Region, which is an important area for cistanche cultivation. The region’s representative climate and environmental conditions make it ideal for cistanche growth. During the field collection process, we utilized high-resolution digital cameras and drones equipped with HD cameras for aerial photography, ensuring images were captured from various angles and heights to comprehensively document the progression of diseases. Specifically, the digital camera used was a Nikon D850 equipped with a macro lens, capable of capturing detailed features of cistanche plants, such as minute changes in leaves and stems. For drones, the DJI Mavic Air 2 was employed, featuring a 48 MP camera providing sufficient detail for large-scale aerial disease monitoring. During the collection process, special attention was given to several major cistanche diseases, including stem rot, root rot, powdery mildew, aphids, and stem-borer damage. Each disease was represented by 1000to 1800 images, ensuring adequate data volume and a comprehensive representation of disease characteristics. The number of images for each pest and disease is shown in Table 1.

To enhance the quality of the image data, we ensured images were taken under adequate lighting conditions and avoided using flash to prevent reflection or overexposure, which could interfere with subsequent image processing and analysis. The collection process followed a standardized protocol. Before each session, the research team conducted a thorough pre-survey of the study area, marking diseased plants and recording their exact locations and growth conditions. During image collection, each plant was photographed from four different directions (front, back, left, and right) to comprehensively document its health status.

Furthermore, for each disease, we provided detailed descriptions, recording images from the early, middle, and late stages of disease progression to enable the model to learn the complete development process of diseases. For example, early-stage stem rot typically manifests as water-soaked brown spots at the stem base, which gradually spread upward, leading to visible decay in the late stage. Root rot primarily affects the root system, causing it to blacken and rot, with severe cases leading to overall plant wilting. Powdery mildew appears as white powdery substances covering the leaf surface, significantly hindering photosynthesis. Aphid and stem-borer damage were identified by observing the quantity of aphids and the perforation patterns on the stems caused by borers. By meticulously documenting these features, we built a comprehensive and detailed image library of cistanche diseases, providing rich learning material for subsequent model training. To further diversify the dataset, images were also sourced online from agricultural research websites and professional forums related to cistanche diseases. Before using these images, we conducted rigorous screening and preprocessing to ensure their authenticity and usability.

### 3.2. Data Augmentation

#### 3.2.1. Basic Augmentation Methods

Data augmentation artificially expands the dataset to enhance model generalization and robustness. Common techniques simulate real-world variations, aiding disease detection under diverse conditions. Rotation diversifies perspectives by rotating an image at a random angle, which was achieved using the transformation matrix:

(9) $$R\left(\theta \right)=\left[\begin{array}{ccc} \cos(\theta ) & -\sin(\theta ) & 0\\ \sin(\theta ) & \cos(\theta ) & 0\\ 0 & 0 & 1 \end{array}\right]$$

Scaling adjusts image size to simulate varying target distances, using

(10) $$S\left(s_{x},\;s_{y}\right)=\left[\begin{array}{ccc} s_{x} & 0 & 0\\ 0 & s_{y} & 0\\ 0 & 0 & 1 \end{array}\right]$$

Cropping removes portions of the image to mimic occlusion, improving model robustness to missing data. Flipping enhances diversity by mirroring images horizontally or vertically, which is represented as

(11) $$F_{h}=\left[\begin{array}{ccc} -1 & 0 & W\\ 0 & 1 & 0\\ 0 & 0 & 1 \end{array}\right],\;F_{v}=\left[\begin{array}{ccc} 1 & 0 & 0\\ 0 & -1 & H\\ 0 & 0 & 1 \end{array}\right]$$

where *W* and *H* are the image width and height. Color jittering adjusts brightness, contrast, and saturation to simulate varying lighting conditions. These augmentation methods enhance the model’s adaptability to diverse environments, improving accuracy and robustness while effectively expanding the dataset.

#### 3.2.2. Mixup, Cutout, Random Erase

**Figure 1:**
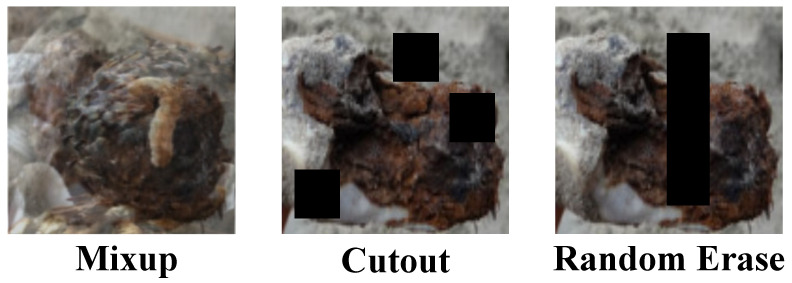
Visualization of different image enhancement methods.

In the machine learning application of cistanche disease detection, in addition to traditional image augmentation methods, more advanced techniques such as Mixup, Cutout, and Random Erase are also widely used to further enhance the model’s generalization ability and robustness, as shown in Figure 1. These methods alter the training data more aggressively, effectively improving the model’s ability to adapt to various complex environmental conditions.

The Mixup method is a technique that generates new training samples by linearly interpolating between two images and their corresponding labels during the training phase. Specifically, given two random training images and their labels, Mixup generates new images and labels using the following formula:

(12) $${\mathrm{Image}}_{\mathrm{new}}=\lambda \times {\mathrm{Image}}_{1}+\left(1-\lambda \right)\times {\mathrm{Image}}_{2}$$

(13) $${\mathrm{Label}}_{\mathrm{new}}=\lambda \times {\mathrm{Label}}_{1}+\left(1-\lambda \right)\times {\mathrm{Label}}_{2}$$

where λ is a value randomly sampled from a Beta distribution Beta(α,α), typically with α ranging from 0.2 to 0.4. This method effectively expands the data distribution, allowing the model to learn to handle features that are synthesized from different images, thereby enhancing its ability to recognize occluded and subtle disease features. The Cutout method involves randomly selecting a region in the training image and setting its pixel values to zero to artificially create occlusion, simulating partial occlusion in real-world environments. The core idea behind this method is to weaken certain parts of the image’s information, forcing the model to search for and use other relevant information in the image for learning and prediction. Its basic operation can be expressed as

(14) $${\mathrm{Image}}_{\mathrm{masked}}=\mathrm{Image}-\mathrm{Mask}$$

where Mask is a matrix of the same size as the image, and the values in the selected occlusion region correspond to the image’s values at those positions, while other positions are set to zero. The Random Erase method is similar to Cutout, but instead of filling the selected image region with zeros, it fills it with randomly generated pixel values. This not only simulates occlusion but also introduces random noise, causing the model to not only learn to identify important disease features but also learn to ignore irrelevant noise. The random erase operation can be described as

(15) $${\mathrm{Image}}_{\mathrm{erased}}=\mathrm{Replace}\left(\mathrm{Image},\;\mathrm{Region},\;\mathrm{Random}\;\mathrm{Pixels}\right)$$

Here, the Replace function replaces the specified Region in an image with RandomPixels, which are randomly generated from a possible pixel value distribution. By applying these data augmentation techniques, the cistanche disease detection model can more effectively handle various challenges encountered in practical applications, such as partial occlusion, image damage, and lighting changes.

### 3.3. Proposed Method

#### 3.3.1. Overview

**Figure 2:**
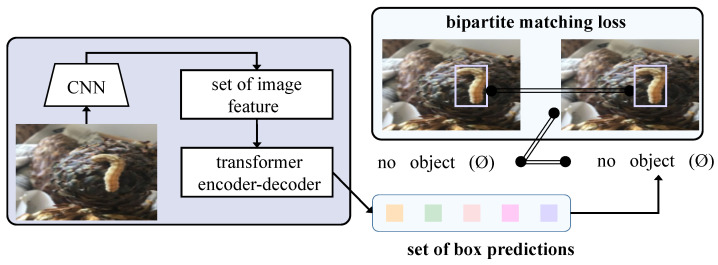
Flowchart of proposed method.

This paper proposes a model structure based on Transformer technology in deep learning, combined with the latest methods in the field of object detection, to design an efficient and accurate framework for detecting cistanche diseases. The entire model process begins with processed image input, passing through multiple modules step by step, ultimately achieving an accurate identification and localization of disease regions, as shown in Figure 2.

After data preprocessing and augmentation, the image data serve as the model input and first pass through a series of convolutional layers for feature extraction. At this stage, the input image is transformed into multi-dimensional feature maps that contain spatial and color information of the image. These feature maps serve as the input to the Transformer model, and the local features extracted through the CNN ensure that the details in the image are effectively preserved. Next, the feature maps extracted by the CNN are passed into the Transformer encoder. The Transformer encoder models the global information of the image and captures long-range dependencies between different regions of the image through the bridging attention mechanism. The bridging attention mechanism introduces a bridging layer in the self-attention computation to connect low-level local features and high-level semantic features. Specifically, this bridging layer fuses low-level and high-level features in a weighted manner after each attention calculation. This design enables the model to more precisely identify disease regions, maintaining high detection accuracy even in cases where the boundaries between disease regions and the background are blurred or where disease features are subtle.

During the training phase of the model, the bridging loss function combines classification and regression losses in a weighted manner with the weighting coefficients dynamically adjusted throughout the training process. This design automatically shifts the focus of the loss function according to the demands of classification and regression tasks at different training stages. Finally, the model outputs the category labels and bounding box coordinates for each target. The entire process ensures that every step from input to output maximizes the effectiveness of each module, enabling the model to quickly and accurately identify and localize disease regions in practical applications. This significantly enhances the efficiency and effectiveness of disease detection in smart agriculture.

#### 3.3.2. Transformer-Based Object Detection Network

**Figure 3:**
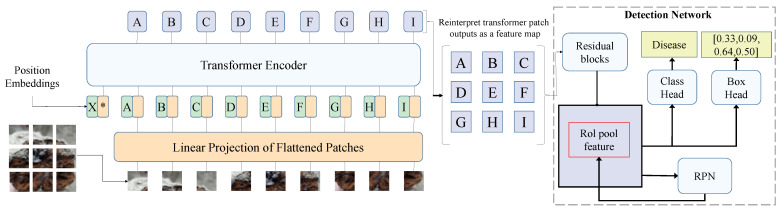
Architecture of transformer-based object detection network. The entire model consists of three main components: the image input module, the Transformer encoder, and the object detection network. First, the input image is divided into multiple small patches and converted into feature representations through the Linear Projection of Flattened Patches. These features are then fed into the Transformer encoder, where they are processed along with Position Embeddings to facilitate global feature learning. The letters A, B, C, D, E, F, G, H, and I in the figure represent different image patches mapped through the Transformer encoder, which are subsequently rearranged into a feature map after undergoing the self-attention mechanism.

In this study, a Transformer-based object detection network is proposed to address the challenges in detecting cistanche diseases, particularly when dealing with complex backgrounds, multiple targets, occlusion, and small objects, as shown in Figure 3. The core of this model is the encoder–decoder structure of Transformer, which effectively captures both global information and local details in images. It enhances feature representation through the self-attention mechanism and ultimately achieves the objective of object detection. Unlike traditional CNNs, the Transformer structure models images globally, capturing long-range dependencies through multi-level self-attention mechanisms, thus better handling complex backgrounds and occlusions.

The encoder transforms the input image’s feature map into a global representation, capturing relationships between different regions of the image. In our model, the encoder consists of multiple layers of self-attention mechanisms and feedforward neural networks. Specifically, the encoder structure comprises *N* layers of self-attention modules, each with a dimension of $d_{\mathrm{model}}=512$ and a per-head dimension of $d_{\mathrm{head}}=64$. This design enables each self-attention head to capture distinct relational information in parallel. In each layer of the self-attention mechanism, the input feature map is weighted and summed with positional encoding-enhanced features to achieve spatial information modeling. The output of each self-attention layer is passed to a feedforward neural network (FFN), which consists of two fully connected layers with a ReLU activation function in between. The FFN enhances the feature representation at each position through nonlinear transformations. Mathematically, the FFN operation can be expressed as

$$\mathrm{FFN}\left(z\right)=\max\left(0,\;zW_{1}+b_{1}\right)W_{2}+b_{2}$$

Here, z is the output of the self-attention mechanism, $W_{1}$ and $W_{2}$ are the weight matrices of the fully connected layers, $b_{1}$ and $b_{2}$ are bias terms, and ReLU is the activation function. This process enables the encoder to perform complex transformations on the input feature map and provide richer feature representations for the decoder. In Figure 3, the letters A, B, C, etc., represent different patch embeddings processed through the Transformer encoder. These patches undergo attention-based feature refinement before being reconstructed into a feature map for the detection network.

The decoder generates each target’s class and bounding box based on the encoder’s output. In our model, the decoder also consists of multiple layers of self-attention modules and introduces the concept of object queries. Object queries are a learned set of vectors with each query corresponding to a specific target. Through interaction with the encoder’s output features, object queries incrementally generate class predictions and bounding box regressions. The decoder’s core structure is similar to the encoder, and it is also based on the multi-head self-attention mechanism. In the decoder, each object query interacts with the encoder’s feature map via self-attention to capture the relationship between targets and the background. After multiple computations, the object queries output the class probabilities and bounding box coordinates for each target. The class and location of each target can be calculated as follows:

$${\hat{y}}_{i}=\mathrm{softmax}\left(q_{i}W_{\mathrm{cls}}\right)$$

$${\hat{b}}_{i}=q_{i}W_{\mathrm{box}}+b_{\mathrm{box}}$$

Here, $q_{i}$ is the object query, $W_{\mathrm{cls}}$ and $W_{\mathrm{box}}$ are the weight matrices for classification and regression, and $b_{\mathrm{box}}$ is the bias term for the bounding box. ${\hat{y}}_{i}$ and ${\hat{b}}_{i}$ are the class probabilities and bounding box coordinates for the target, respectively. The output of the decoder has the same dimension as the input feature map, but each query focuses only on one target. Thus, the decoder generates the predictions for each target individually through object queries. This mechanism allows the decoder to identify targets sequentially in global information, adapting to multi-target detection in complex scenarios.

Through the above design, the Transformer-based object detection network effectively leverages the advantages of self-attention mechanisms and object queries to achieve the precise localization and classification of multiple targets in an image. First, the Transformer architecture captures global information, avoiding the limitations of local receptive fields in traditional CNNs. Second, the introduction of the bridging attention mechanism enables the model to maintain global contextual information while focusing on local details, enhancing its sensitivity to small objects and occlusion. This design shows significant advantages in handling complex backgrounds, multiple overlapping targets, and small objects in cistanche disease detection. In practical applications, traditional object detection methods often experience degraded accuracy due to complex backgrounds, varying target sizes, or poor image quality. The introduction of a Transformer-based object detection network mitigates these challenges by providing a robust, accurate, and efficient solution capable of stable operation in complex environments. This contributes reliable technical support for precision agriculture and intelligent disease management.

#### 3.3.3. Bridging Attention Mechanism

**Figure 4:**
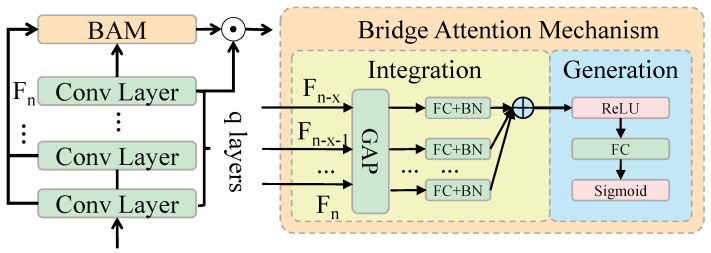
Architecture of bridge attention mechanism.

In traditional Transformer architectures, the self-attention mechanism captures global information by calculating the similarity between input features. While this method excels at modeling long-range dependencies and global context, it is not specifically designed to handle the relationship between low-level details and high-level semantics. In object detection tasks, particularly for cistanche disease detection, detailed features such as the morphology and color variations of disease spots are critical for accurate detection. However, the self-attention mechanism may overly focus on global information, neglecting the representation of these detailed features. The core idea of the bridging attention mechanism is to introduce weighted fusion between low-level features and high-level semantic features on top of the self-attention mechanism, as shown in Figure 4. In each layer of the self-attention mechanism, an additional fusion step is incorporated, which combines features from different levels through weighted integration, thereby enhancing the model’s focus on local details without losing the ability to model global semantics.

Specifically, the bridging attention mechanism combines low-level features (e.g., detailed information extracted via CNNs) and high-level features (e.g., global information from the Transformer encoder) in a weighted manner. This design enables the model to simultaneously attend to detailed parts of the image and the overall background. This is particularly useful for maintaining high accuracy when dealing with complex backgrounds, occlusions, and small targets. In the bridging attention mechanism, the input features are first processed through the self-attention mechanism to obtain weighted representations for each position, which are then fused with the low-level and high-level features in a weighted manner. The mathematical representation is as follows:

$$Z_{\mathrm{bridged}}=\mathrm{Attention}\left(Q,\;K,\;V\right)+\alpha \cdot F_{\mathrm{low}}+\beta \cdot F_{\mathrm{high}}$$

Here, Q, K, and V denote the query, key, and value inputs, respectively. α and β are dynamically learned weighting coefficients, while $F_{\mathrm{low}}$ and $F_{\mathrm{high}}$ represent the low-level and high-level features, respectively. The weighting coefficients α and β control the fusion ratio of low-level details and high-level semantics. During training, the model automatically adjusts these coefficients based on the complexity of the input image to find the optimal feature fusion strategy. This design effectively enhances the model’s ability to identify disease regions by integrating low-level and high-level features.

Low-level details help the model capture subtle changes in disease spots, while high-level semantic information aids in identifying potential distractions in the background. Through this weighted fusion, the bridging attention mechanism effectively avoids the self-attention mechanism’s tendency to overemphasize background noise, ensuring an accurate recognition of disease features. To implement the bridging attention mechanism, the proposed Transformer-based object detection network includes multiple encoder and decoder layers. Each encoder layer has a dimension of $d_{\mathrm{model}}=512$, and each self-attention head has a dimension of $d_{\mathrm{head}}=64$. Within each layer, the input feature map undergoes weighted summation through multi-head self-attention, producing enhanced feature representations. These representations are then passed to an FFN for further processing, resulting in the final feature output. In the decoder, the object query dimension is set to $d_{\mathrm{query}}=512$, matching the encoder’s output dimension. Object queries interact with the encoder’s feature maps via multi-head self-attention to generate object classification and bounding box regression results. In each decoder layer, the bridging attention mechanism is also applied to fuse low-level details and high-level semantic features, enhancing the accuracy of object localization and classification. Each layer of the bridging attention mechanism consists of the following steps:

1. The input feature map $F_{\mathrm{input}}$ is first processed through the self-attention mechanism to generate weighted representations for each position.
2. Low-level features $F_{\mathrm{low}}$ and high-level features $F_{\mathrm{high}}$ are then fused with weights and combined with the output of the self-attention mechanism to produce the final bridging features $Z_{\mathrm{bridged}}$.
3. Finally, the bridging features are processed through a feedforward neural network to generate the final object classification and regression results.

The bridging attention mechanism addresses the limitations of traditional self-attention mechanisms in object detection tasks, particularly in terms of accuracy and robustness. Mathematically, the superiority of the bridging attention mechanism can be demonstrated by calculating the difference between the weighted fused bridging features and the original features. Assume that the input feature map of the image is $F_{\mathrm{input}}$ with dimensions H×W×C, where *H* is the image height, *W* is the image width, and *C* is the number of channels. The low-level features $F_{\mathrm{low}}$ and high-level features $F_{\mathrm{high}}$ extracted by the CNN represent detailed and semantic information, respectively. In traditional Transformers, these features are processed independently. However, in the bridging attention mechanism, the fusion of $F_{\mathrm{low}}$ and $F_{\mathrm{high}}$ ensures that the model maintains sensitivity to local details while achieving global modeling. Through the above design, the bridging attention mechanism plays the following key roles in the model:

1. Enhancing detail information: Low-level features help the model focus on subtle changes in disease spots, which is critical for detecting cistanche diseases.
2. Improving global semantic representation: High-level semantic features provide global background information, aiding the model in understanding the relationship between targets and the background.
3. Increasing the accuracy of small and occluded target detection: By fusing low-level and high-level information, the model is better equipped to handle challenges arising from occluded targets and varying target sizes.

Thus, the bridging attention mechanism significantly improves the accuracy of object detection by enhancing the ability to learn local details while maintaining global contextual modeling. It demonstrates robustness and accuracy, particularly in complex backgrounds and small-target scenarios.

#### 3.3.4. Bridging Loss Function

In object detection tasks, the design of the loss function directly determines the learning effectiveness and final performance of the model. Traditional object detection methods typically divide the loss into classification loss (e.g., cross-entropy loss) and regression loss (e.g., smooth L1 loss) for predicting target categories and bounding box locations, respectively. However, this design faces two major issues in practical applications: first, the optimization goals of classification and regression are different and difficult to coordinate; second, fixed loss weights cannot dynamically adapt to the needs of different training stages. To address these problems, this paper proposes a bridging loss function, which significantly improves the model’s performance in complex object detection tasks through dynamic weight adjustment and balanced multi-task loss. Its core idea is to introduce a bridging term $L_{\mathrm{bridging}}$, establishing a dynamic balance between classification and regression while assigning higher optimization weights to difficult-to-detect targets. The overall form of the bridging loss function is as follows:

(16) $$L_{\mathrm{total}}=\lambda_{\mathrm{cls}}L_{\mathrm{cls}}+\lambda_{\mathrm{reg}}L_{\mathrm{reg}}+\lambda_{\mathrm{bridging}}L_{\mathrm{bridging}}$$

In Equation (16):

(1) $L_{\mathrm{cls}}$ is the classification loss, which is defined using the cross-entropy loss function:
  (17) $$L_{\mathrm{cls}}=-\frac{1}{N}\sum_{i=1}^{N}\left[y_{i}\log\left(p_{i}\right)+\left(1-y_{i}\right)\log\left(1-p_{i}\right)\right]$$
  where $y_{i}$ is the target class label, and $p_{i}$ is the predicted class probability.
(2) $L_{\mathrm{reg}}$ is the regression loss, which is defined using the smooth L1 loss function:
  (18) $$L_{\mathrm{reg}}=\frac{1}{N}\sum_{i=1}^{N}\mathrm{SmoothL}1\left(b_{i}-{\hat{b}}_{i}\right)$$
  The smooth L1 loss is defined as
  (19) $$\mathrm{SmoothL}1\left(x\right)=\left\{\begin{array}{cc} 0.5x^{2} & \mathrm{if}\;|x|<1,\\ |x|-0.5 & \mathrm{otherwise}. \end{array}\right.$$
(3) $L_{\mathrm{bridging}}$ is the bridging term, which is used to dynamically balance classification and regression losses while enhancing the weight of difficult examples:
  (20) $$L_{\mathrm{bridging}}=\frac{1}{N}\sum_{i=1}^{N}w_{i}\cdot \left|L_{\mathrm{cls},i}-L_{\mathrm{reg},i}\right|$$
  where $w_{i}$ is the weight for difficult examples, which is defined as
  (21) $$w_{i}=1+\gamma \cdot {\mathrm{IoU}}_{\mathrm{low}}$$
  Here, ${\mathrm{IoU}}_{\mathrm{low}}$ is the low IoU value between the current predicted box and the ground truth box, which is used to identify targets that are difficult to localize; γ is a scaling parameter to amplify the impact of difficult examples.

The bridging loss function improves classification and regression precision in object detection tasks by dynamically balancing and optimizing difficult examples. Mathematically, the introduction of the bridging term $L_{\mathrm{bridging}}$ helps reduce the discrepancy between classification loss and regression loss, thereby improving the overall performance of the model. Let $L_{\mathrm{cls}}^{*}$ and $L_{\mathrm{reg}}^{*}$ represent the expected values of classification and regression losses, respectively. Traditional loss functions aim to minimize $L_{\mathrm{cls}}+L_{\mathrm{reg}}$, but this may lead to inconsistent optimization between classification and regression tasks. In the bridging loss function, $L_{\mathrm{bridging}}$ enforces the discrepancy between classification and regression losses to approach zero, i.e.,

(22) $$\lim_{t\to \infty }L_{\mathrm{bridging}}\to 0\Rightarrow L_{\mathrm{cls}}^{*}\approx L_{\mathrm{reg}}^{*}$$

Through this mechanism, the model can automatically balance classification and regression tasks, avoiding excessive interference from a single task on the overall optimization.

## 4. Results and Discussion

### 4.1. Experimental Setup

#### 4.1.1. Hardware and Software Platform

In this experiment, high-performance computing hardware was selected to support the training and testing of deep learning models. The specific hardware configuration includes a server equipped with an NVIDIA Tesla V100 GPU, which has 32 GB of VRAM, providing sufficient parallel processing power to handle large-scale image data and complex model computations. Additionally, the server is configured with 128 GB of RAM and an Intel Xeon E5-2686 v4 @ 2.30GHz CPU, ensuring efficient data processing and model training. This hardware setup not only shortens the model training time but also improves the efficiency of the experiment, allowing the model to iterate and optimize in a shorter time frame.

On the software platform side, to fully utilize the hardware resources and enhance the flexibility and efficiency of the research, Python was chosen as the primary development tool, taking advantage of its powerful library support for deep learning model design and experimentation. The primary frameworks used in the experiment were TensorFlow and Keras, which provide rich APIs and highly optimized backend support, making the construction, training, and testing of models more straightforward and efficient. We also used the NumPy and Pandas libraries for data preprocessing and analysis as well as Matplotlib and Seaborn for result visualization. Additionally, to ensure the reproducibility of the experiment and version control, the experimental code was hosted on the Git platform, and all dependencies and environments were managed using Docker containers. This approach allows the experiment to be quickly deployed and run across different hardware environments, ensuring the consistency and accuracy of the results.

#### 4.1.2. Dataset Split and Hyperparameters

In this study on cistanche disease detection, the entire dataset of cistanche disease images was divided into three parts: 70% of the data was used as the training set for model learning and parameter adjustment; 15% of the data was used as the validation set for hyperparameter tuning and to prevent overfitting; the remaining 15% was used as the test set to evaluate the final performance of the model. Additionally, to optimize model performance and ensure the stability of the training process, we carefully adjusted parameters such as learning rate, batch size, and training epochs. The learning rate was initially set to 0.001, with an exponential decay strategy applied to optimize the training process, and the decay rate was set to 0.95, updating the learning rate after each epoch. The batch size was set to 32, which was determined based on GPU memory capacity and model complexity to ensure the efficient use of computational resources while maintaining good training dynamics. The number of training epochs was set to 100, which is sufficient to allow the model to converge under various parameter configurations. To further ensure the accuracy and fairness of the model evaluation, this study adopted five-fold cross-validation. In five-fold cross-validation, the dataset is randomly divided into five non-overlapping subsets. In each experiment, four subsets are used for training, and the remaining subset is used for testing. This method effectively reduces the impact of data partition randomness on the model evaluation results, providing a more realistic reflection of the model’s generalization ability on unseen data.

#### 4.1.3. Evaluation Metrics

In the research on cistanche disease detection, several evaluation metrics were adopted, including accuracy, precision, recall, Frames Per Second (FPS) and mean Average Precision (mAP) at different IoU thresholds (mAP@50 and mAP@75). These metrics collectively reflect the model’s performance in disease detection tasks, helping us understand the model’s strengths and limitations from different perspectives. Accuracy is the most straightforward performance metric, reflecting the model’s ability to correctly identify whether a disease is present in an image. The accuracy is calculated as the number of correct predictions divided by the total number of samples. Precision measures the proportion of predicted disease samples that are actually diseases, which is a key indicator for evaluating the accuracy of the model’s predictions. Recall, on the other hand, focuses on the proportion of actual disease samples that are correctly identified by the model, reflecting the model’s ability to capture disease samples. mAP is a commonly used performance metric in object detection, which calculates the average precision (AP) at different recall levels and evaluates the model’s requirement for target localization accuracy by considering different IoU thresholds. mAP@50 and mAP@75 represent the mAP values at IoU thresholds of 0.5 and 0.75, respectively, reflecting the model’s performance in target localization accuracy under different levels of strictness. In addition to accuracy-based evaluation, FPS is introduced as a critical metric to assess the model’s inference speed. FPS measures the number of frames the model can process per second, which is crucial for real-time disease detection applications. A higher FPS indicates better computational efficiency, making the model more suitable for deployment in large-scale agricultural monitoring systems. The mathematical formulas for these metrics are as follows:

(23) $$\mathrm{Accuracy}=\frac{\mathrm{TP}+\mathrm{TN}}{\mathrm{TP}+\mathrm{TN}+\mathrm{FP}+\mathrm{FN}}$$

(24) $$\mathrm{Precision}=\frac{\mathrm{TP}}{\mathrm{TP}+\mathrm{FP}}$$

(25) $$\mathrm{Recall}=\frac{\mathrm{TP}}{\mathrm{TP}+\mathrm{FN}}$$

(26) $$\mathrm{mAP}=\frac{1}{n}\sum_{t=1}^{n}{\mathrm{AP}}_{t}$$

(27) $$\mathrm{FPS}=\frac{\mathrm{Number}\;\mathrm{of}\;\mathrm{Processed}\;\mathrm{Images}}{\mathrm{Total}\;\mathrm{Processing}\;\mathrm{Time}\;\left(\mathrm{Seconds}\right)}$$

where TP represents the number of true positives, i.e., diseases correctly identified; TN represents the number of true negatives, i.e., non-diseases correctly classified; FP represents the number of false positives, i.e., non-diseases misclassified as diseases; FN represents the number of false negatives, i.e., diseases misclassified as non-diseases. The parameter *n* represents the number of different IoU thresholds, and ${\mathrm{AP}}_{t}$ is the average precision computed at the *t*-th IoU threshold. By evaluating the model using these metrics, we can gain a comprehensive understanding of the model’s performance in cistanche disease detection, identify potential issues in real-world applications, and optimize the model design accordingly.

### 4.2. Baseline

In the study of cistanche disease detection, to comprehensively assess the performance of the proposed model, we selected several popular and representative object detection models as baselines for comparison, including YOLO v8 [47], YOLO v9 [48], DETR [49], and Faster R-CNN [50]. The YOLO series of models are widely used in real-time object detection tasks due to their fast detection speed, with v8 and v9 being the latest versions of the series, offering improved detection accuracy and faster processing speeds. This makes them highly suitable for practical applications that require rapid responses. DETR introduces the Transformer architecture to object detection, achieving precise target localization and classification through global self-attention mechanisms. This method performs excellently in handling complex scenes and multi-object detection tasks. Faster R-CNN, as a classic two-stage object detection model, first generates candidate regions using a region proposal network, which is followed by detailed classification and bounding box regression. It is highly reliable in terms of detection accuracy, especially for research fields requiring high precision. By comparing our model with these baseline models, we can evaluate the performance of the new model in terms of speed, accuracy, and robustness from multiple dimensions, further identifying and optimizing potential shortcomings to ensure its competitiveness and effectiveness in practical applications.

### 4.3. Pest and Disease Detection Results

The primary objective of this experiment is to compare the performance of different models in the cistanche pest and disease detection task, evaluating their classification and localization capabilities across multiple metrics, including precision, recall, accuracy, Frames Per Second (FPS), and mean Average Precision (mAP@50 and mAP@75), as shown in Table 2. These metrics comprehensively assess the performance of each model under various pest and disease characteristics, providing data to support the selection of the optimal detection scheme. According to the results in the table, the traditional two-stage model Faster-RCNN exhibits relatively low performance across metrics but maintains balanced results, showcasing its robust region proposal capabilities. However, due to the complexity and computational demands of its two-stage structure, it falls short in recall, high IoU scenarios (mAP@75), and inference speed, achieving the lowest FPS among all models. DETR improves global information modeling significantly by introducing the Transformer self-attention mechanism, achieving slight enhancements in precision and recall. However, its adaptability to small objects and complex backgrounds remains a limitation, resulting in slightly subpar localization accuracy. Additionally, DETR’s reliance on bipartite matching for object queries leads to lower FPS compared to single-stage models.

YOLO models leverage their efficient single-stage architecture and innovative multi-scale feature fusion mechanism, outperforming Faster-RCNN and DETR across all metrics. Among them, YOLO v9 demonstrates outstanding precision, recall, and mAP scores, reflecting its advanced feature extraction and context enhancement capabilities. Furthermore, YOLO models achieve significantly higher FPS values, highlighting their efficiency for real-time applications. The proposed method achieves the highest scores across all accuracy-related metrics, notably reaching precision and recall levels of 0.95 and 0.92, respectively, indicating its comprehensive improvement in extracting and localizing pest and disease features. In addition, it maintains a competitive FPS compared to YOLO models, demonstrating a well-balanced trade-off between accuracy and computational efficiency, making it suitable for real-time agricultural monitoring applications.

From a theoretical perspective, the superior performance of the proposed method is primarily attributed to the introduction of the bridging attention mechanism and the bridging loss function, specifically designed for agricultural scenarios. Mathematically, the bridging attention mechanism enhances sensitivity to disease regions in complex backgrounds by the weighted fusion of low-level detail features and high-level semantic features. Compared to Faster-RCNN’s region proposal approach and DETR’s global self-attention mechanism, the bridging attention mechanism strikes a better balance between capturing local details and maintaining global context, significantly improving mAP@50 and mAP@75. Furthermore, the bridging loss function dynamically adjusts the weights of classification and regression losses, addressing inconsistencies in optimization objectives between tasks in traditional loss functions. Theoretically, this design reduces model bias caused by class imbalance and target scale differences, enhancing accuracy in high IoU scenarios (i.e., mAP@75). Compared to the YOLO series, the proposed model incorporates a semantic enhancement module based on multi-scale feature fusion, improving precision in detecting small targets and complex disease morphologies. Despite incorporating Transformer-based modules, the proposed method maintains an FPS of 47, close to that of YOLO v9, demonstrating that it achieves a favorable trade-off between computational efficiency and detection accuracy. This is a critical factor for its overall superiority in precision and recall compared to YOLO v9 while maintaining real-time processing capability. In conclusion, the excellent performance of the proposed method not only demonstrates the effectiveness of the model structure and optimization strategy but also provides reliable technical support for precision pest and disease detection in smart agriculture. By achieving high detection accuracy alongside competitive inference speed, the proposed model is well-suited for real-time agricultural monitoring and large-scale automated disease detection.

### 4.4. Results Analysis

The experiment’s primary objective is to evaluate the proposed method’s performance in detecting various pests and diseases, analyzing the model’s classification and localization capabilities across different types of diseases, including stem rot, root rot, powdery mildew, aphids, and stem borers, as shown in Table 3. The results demonstrate that for stem rot, the model exhibits the best performance, with all metrics reaching or approaching 0.96, indicating its strong detection capability in scenarios with evident and well-defined disease features. For root rot and powdery mildew, the detection performance is slightly lower than that for stem rot, but precision, recall, and mAP metrics remain high. This indicates that the model maintains stable performance even in scenarios where the details are more complex or the disease distribution is broader. For aphids and stem borers, due to their subtle features and smaller target size, the model’s precision and recall are slightly lower. However, the small difference between mAP@50 and mAP@75 suggests that the model’s overall performance in small-target detection tasks remains acceptable. Additionally, FPS (Frames Per Second) is introduced as a key evaluation metric to assess the model’s inference speed across different disease categories. Since the detection complexity varies depending on disease morphology and distribution, FPS provides insight into how computational efficiency is affected in different cases. Diseases with well-defined visual characteristics (e.g., stem rot) generally result in higher FPS, whereas small and complex diseases (e.g., aphids) may lead to slightly lower FPS due to increased computational demand.

From a mathematical perspective, the key to the excellent results achieved by the proposed method lies in the effective fusion of low-level and high-level features and the dynamic balancing strategy of the bridging loss function. The high detection accuracy for stem rot is primarily attributed to the bridging attention mechanism’s ability to effectively integrate local details and global context, enabling the model to accurately capture the morphological characteristics of the disease region. Mathematically, this fusion is achieved through dynamically adjusting the feature weights α and β, ensuring that the proportion of feature fusion aligns closely with the practical task requirements. For root rot and powdery mildew, where the disease is distributed at different depths or locations, the model must handle multi-scale features simultaneously. This is facilitated by the improvements made to the multi-head attention mechanism within the Transformer structure, allowing each attention head to capture feature variations at different scales, thereby enhancing mAP performance. For small-target diseases like aphids and stem borers, where target regions are small and detection difficulty is high, the bridging loss function is specifically designed to increase the weights of difficult examples. Particularly, the IoU-based dynamic weighting strategy targets disease targets that are difficult to locate. Consequently, even in these challenging detection scenarios, the proposed method maintains balanced performance and high robustness. These results validate the proposed method’s efficiency and broad applicability in handling different disease types and complex scenarios.

### 4.5. Confusion Matrix Analysis

**Figure 5:**
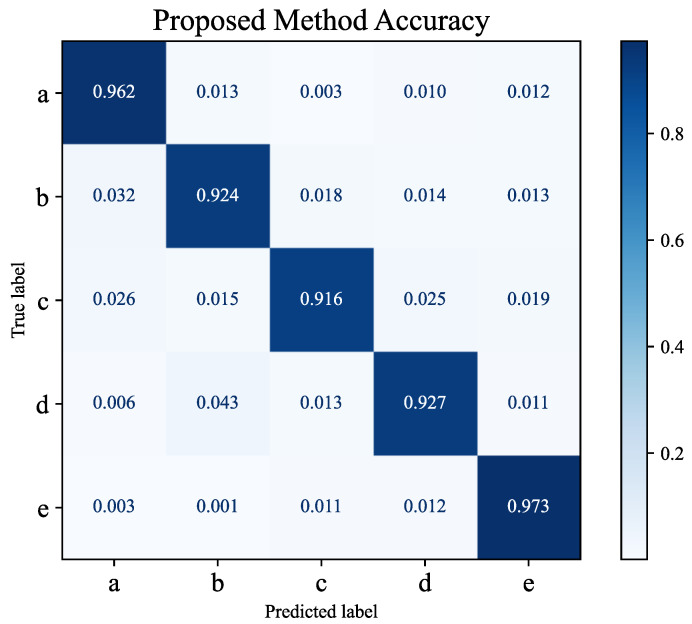
Confusion matrix: a is powdery mildew; b is stem rot; c is root rot; d is aphids; e is stem-borer damage.

The primary objective of this experiment is to analyze the classification accuracy and error rates of the model across different pest and disease categories through confusion matrix analysis, providing a comprehensive evaluation of the model’s performance in practical detection tasks. The confusion matrix results indicate that the model achieves the highest classification accuracy for stem rot and root rot with correct recognition rates nearing 100%, as shown in Figure 5. However, a slight decline in classification performance is observed for powdery mildew and aphids, which is primarily due to occasional misclassification into other categories.

The misclassification may be attributed to the similarity of features between certain diseases and the complexity of the background. For instance, powdery mildew in some scenarios resembles leaf texture changes under natural lighting, while aphids, being small in size, are easily obscured by background noise. Theoretically, confusion matrix analysis reflects the model’s limitations in handling overlapping features among categories while also validating the effectiveness of the proposed bridging attention mechanism and bridging loss function. The bridging attention mechanism enhances the model’s sensitivity to local details by the weighted fusion of low-level and high-level features, enabling excellent performance in scenarios with distinct disease features. However, the occurrence of misclassification in scenarios with ambiguous disease features or complex backgrounds highlights the need to further optimize the model’s robustness in specific cases. Moreover, insights from the misclassification analysis provided by the confusion matrix can guide targeted improvements in data augmentation strategies, enhancing the model’s adaptability to overlapping features and complex backgrounds. These findings offer clear directions for further model optimization. In conclusion, the confusion matrix analysis not only validates the model’s exceptional performance in most scenarios but also identifies areas for improvement, laying a solid foundation for further research in precision pest and disease detection in smart agriculture.

### 4.6. Ablation Study on Different Attention Mechanisms

The main objective of this experiment is to analyze the impact of different attention mechanisms on the performance of the cistanche disease detection task through ablation studies, thereby validating the effectiveness of the proposed bridging attention mechanism, as shown in Table 4. Three different attention mechanisms were compared in the experiment: standard self-attention, CBAM, and the proposed bridging attention mechanism. The standard self-attention mechanism showed relatively poor overall performance with mAP@50 and mAP@75 scores of 0.74 and 0.72, respectively. While it demonstrated some advantages in modeling long-range dependencies, it lacked effective focus on local detail features. CBAM significantly improved all metrics by combining spatial and channel attention mechanisms, achieving an mAP@50 score of 0.84, highlighting its strong capability in enhancing local features. However, compared to the bridging attention mechanism, CBAM still showed limitations in global information modeling. The bridging attention mechanism achieved the best performance across all metrics, with mAP@50 and mAP@75 scores of 0.92 and 0.90, respectively, indicating its superiority in balancing global information and local details.

From a mathematical perspective, the superior results of the bridging attention mechanism can be attributed to its dynamic weighted fusion of low-level detail features and high-level semantic features. In the standard self-attention mechanism, input features are processed through dot-product operations among queries Q, keys K, and values V to generate attention weights, capturing long-range dependencies. However, this approach pays insufficient attention to local details, leading to suboptimal feature representation. CBAM enhances $F_{\mathrm{input}}$ by introducing spatial attention and channel attention, which improve features through spatial weighting $F_{\mathrm{spatial}}$ and channel weighting $F_{\mathrm{channel}}$. However, CBAM’s ability to capture global information remains limited, which explains the ceiling in its performance. The bridging attention mechanism introduces a bridging layer to dynamically fuse self-attention outputs $Z_{\mathrm{attention}}$ with low-level detail features $F_{\mathrm{low}}$ and high-level semantic features $F_{\mathrm{high}}$, adjusting the balance between local and global information. This fusion optimizes feature representation in theory, enabling the model to exhibit greater robustness when handling complex backgrounds, small targets, and ambiguous disease features. The significant improvement in experimental results confirms the broad applicability and high efficiency of the bridging attention mechanism in agricultural disease detection tasks. In summary, the bridging attention mechanism not only theoretically addresses the limitations of existing attention mechanisms but also empirically validates its performance advantages in disease detection tasks through ablation experiments, providing critical technical support for precision disease management in smart agriculture.

## 5. Conclusions

This study addresses the significant agricultural challenge of pest and disease detection in cistanche cultivation, proposing a Transformer-based object detection network complemented by an innovative bridging attention mechanism and bridging loss function. As a critical medicinal plant, cistanche faces severe pest and disease threats during its cultivation and production. Accurate and efficient detection technologies are essential for improving yield and quality. Traditional methods often struggle with complex backgrounds and diverse disease features. To tackle this issue, this study constructs a Transformer-centered model framework that integrates global information and local details, significantly enhancing detection performance. Furthermore, the method’s applicability extends beyond cistanche disease detection, demonstrating potential for broader agricultural applications.

The contributions of this work are threefold. First, the design and validation of the bridging attention mechanism effectively overcome the limitations of traditional attention mechanisms in capturing local information by dynamically fusing low-level details and high-level semantics. This mechanism demonstrates exceptional performance, particularly in scenarios involving small targets and complex backgrounds. Second, the proposed bridging loss function dynamically balances classification and regression losses, optimizing multi-task training synergy and improving robustness against class imbalance and target scale variations. Third, extensive experiments validate the model’s performance with the bridging attention-based model achieving optimal results: an average accuracy of 0.93, a precision of 0.95, a recall of 0.92, and mAP@50 and mAP@75 scores of 0.92 and 0.90, respectively. Compared to traditional self-attention mechanisms and CBAM modules, the proposed method achieves significant performance improvements. Additionally, this study highlights the method’s scalability by demonstrating its adaptability to various agricultural disease detection tasks, emphasizing its potential for large-scale deployment.

The experimental results demonstrate that this approach effectively addresses pest and disease detection tasks in complex agricultural scenarios, providing a reliable solution for precision disease management in smart agriculture. Given the method’s promising performance, its application can be expanded to other crops affected by diverse diseases, including wheat, maize, and rice. Future research will focus on integrating the model with high-throughput agricultural monitoring systems, enabling real-time and large-scale disease surveillance. This study not only provides technical support for the sustainable development of cistanche cultivation but also offers new insights and practical approaches for intelligent detection technologies in the agricultural sector.

## Figures and Tables

**Table 1:** Number of images for different diseases and pests before and after enhancement.

Disease	Raw Data Number	Enhancement Number
Powdery Mildew	1092	9828
Stem Rot	1781	16,029
Root Rot	1267	11,403
Aphids	1514	13,626
Stem-Borer Damage	1339	12,051

**Table 2:** Pest and disease detection results.

Model	Precision	Recall	Accuracy	mAP@50	mAP@75	FPS
Faster-RCNN	0.84	0.80	0.82	0.82	0.81	21
DETR	0.87	0.83	0.85	0.84	0.83	18
YOLO v8	0.90	0.86	0.88	0.87	0.85	45
YOLO v9	0.92	0.89	0.90	0.90	0.89	50
Proposed Method	0.95	0.92	0.93	0.92	0.90	47

**Table 3:** Performance analysis of different pests and diseases using the proposed method.

Disease	Precision	Recall	Accuracy	mAP@50	mAP@75
Stem Rot	0.97	0.94	0.96	0.95	0.94
Root Rot	0.96	0.93	0.94	0.94	0.93
Powdery Mildew	0.95	0.92	0.93	0.93	0.92
Aphids	0.93	0.90	0.92	0.92	0.91
Stem Borers	0.92	0.89	0.90	0.91	0.90

**Table 4:** Ablation study on different attention mechanisms.

Attention Mechanism	Precision	Recall	Accuracy	mAP@50	mAP@75
Standard Self-Attention	0.77	0.71	0.74	0.74	0.72
CBAM	0.85	0.81	0.83	0.84	0.83
Bridging Attention	0.95	0.92	0.93	0.92	0.90

## Data Availability

The data presented in this study are available on request from the corresponding author.
